# Effect of elastomeric urethane monomer on physicochemical properties and shrinkage stress of resin composites

**DOI:** 10.1590/0103-6440202305475

**Published:** 2023-10-27

**Authors:** Mário Alexandre Coelho Sinhoreti, Lucas de Oliveira Tomaselli, Mateus Garcia Rocha, Dayane Oliveira, Jean-François Roulet, Saulo Geraldeli

**Affiliations:** 1 Department of Restorative Dentistry, Dental Materials Division, Piracicaba Dental School, University of Campinas, Piracicaba, SP, Brazil.; 2 Restorative Dental Science Department, Operative Dentistry Division, College of Dentistry, University of Florida, Gainesville, FL, USA.; 3 Division of Biomedical Materials, School of Dental Medicine, East Carolina University, Greenville, NC, USA.

**Keywords:** resin composite, elastomeric methacrylate monomer, mechanical properties, shrinkage stress

## Abstract

This study aimed to evaluate the effect of an elastomeric urethane monomer (Exothane-24) in different concentrations on physicochemical properties, gap formation, and polymerization shrinkage stress of experimental resin composites. All experimental composites were prepared with 50 wt.% of Bis-GMA and 50 wt.% of TEGDMA, to which 0 wt.% (control), 10 wt.%, 20 wt.%, 30 wt.%, and 40 wt.% of Exothane-24 were added. Filler particles (65 wt.%) were then added to these resin matrixes. Ultimate tensile strength (UTS: n = 10), flexural strength (FS: n = 10), flexural modulus (FM: n = 10), hardness (H: n = 10), hardness reduction (HR: n = 10), degree of conversion (DC: n = 5), gap width (GW: n = 10), and polymerization shrinkage stress in Class I (SS-I: n = 10) and Class II (SS-II: n = 10) simulated configuration. All test data were analyzed using one-way ANOVA and Tukey’s test (α = 0.05; β= 0.2). Exothane-24 in all concentrations decreased the H, HR, DC, GW, SS-I, and SS-II (p < 0.05) without affecting the UTS, and FS (p > 0.05). Reduction in FM was observed only in the Exothane 40% and 30% groups compared to the control (p < 0.05). Exothane-24 at concentrations 20% and 30% seems suitable since it reduced GW and polymerization SS without affecting the properties of the composite resins tested, except for H.

## Resumo

Este estudo teve como objetivo avaliar o efeito de um monômero elastomérico de uretano (Exothane-24) em diferentes concentrações em propriedades físico-químicas, formação de fenda e tensão de contração de polimerização de resinas compostas experimentais. Todos os compósitos experimentais foram preparados com 50% em peso de Bis-GMA e 50% em peso de TEGDMA, nos quais 0% (controle), 10%, 20%, 30% e 40% em peso de Exothane-24 foram adicionados. Partículas de carga (65% em peso) foram então adicionadas as matrizes resinosas. Resistência coesiva (RC: n = 10), resistência à flexão (RF: n = 10), módulo de flexão (MF: n = 10), dureza (D: n = 10), redução de dureza (RD: n = 10), grau de conversão (GC: n = 5), largura de fenda (LF: n = 10) e tensão de contração de polimerização em simulações de cavidades Classe I (TC-I: n = 10) e Classe II (TC-II: n = 10). Todos os dados do teste foram analisados usando *one-way* ANOVA e teste de Tukey (α = 0,05; β = 0,2). O Exothane-24 em todas as concentrações diminuiu a D, RD, GC, LF, TC-I e TC-II (p < 0,05) sem afetar o RC e RF (p > 0,05). A redução da MF foi observada apenas nos grupos Exothane 40% e 30% em relação ao controle (p < 0,05). O Exothane-24 nas concentrações de 20% e 30% pareceu ser adequado, pois reduziu LF e TC de polimerização sem afetar as propriedades das resinas compostas testadas, exceto para D.

## Introduction

Secondary caries is considered the most common reason for failures of resin-based composite restorations. Despite many advancements in resin composite technologies, their monomers still cause polymerization shrinkage stress at the interface between the restorative material and the dental substrate (dentin or enamel), leading to cusp deflection, postoperative sensitivity, and gap formation ^1^. Incremental insertion techniques have been used to mitigate the complications caused by polymerization shrinkage stress ^2^ ^,^ ^3^. However, resin composite monomers require further improvements, and the effect of resin composite resin matrix on the polymerization shrinkage stress continues to be an important clinical concern ^2^.

Alternative monomers to bisphenol-A glycidyl dimethacrylate (Bis-GMA), triethylene glycol dimethacrylate (TEGDMA), and urethane dimethacrylate (UDMA) have been proposed to reduce polymerization shrinkage stress without affecting the physicochemical properties of the resin composites ^4^. One alternative monomer is the elastomeric urethane methacrylate, commercially known as Exothane^TM^ Elastomer (Esstech, Inc.).

Among the types of Exothane^TM^, Exothane-24 has physicochemical properties that seem more suitable than UDMA and BisGMA, monomers commonly found in resin-based composites (5, 6). Based on its molecular structure ^6^, Exothane-24 might increase the mobility and the relaxation capacity of the forming polymer network due to the size of its molecule (682.85 g/mol of molecular weight) and reduces the polymerization shrinkage effect by dissipating the stress on the elastomeric bonds ^7^. Rocha et al. ^6^ performed a chemical analysis of Exothane- 24 for the first time and revealed that it is an elastomeric urethane tetramethyl methacrylate monomer, having a chemical structure based on cyclic aliphatic chains with urethane bonding structures in the center of the molecule and elastomeric polyether bonds close to the methacrylate functional groups with four sites for vinyl polymerization. Exothane-24 has physical properties - great elongation (5%) and elastic modulus (980 N/mm^2^) - which might also favor the mobility and relaxation capacity of the polymer network during formation ^8^.

Although there are many speculations on the use of Exothane monomers on resin composites, the effect of Exothane-24 on the mechanical and chemical properties of resin composites is still few studied. Therefore, this study aimed to evaluate the effect of Exothane-24 in different concentrations on the physicochemical properties, gap formation, and polymerization shrinkage stress of experimental resin composites. The null hypotheses were: ^1^ Exothane-24 would not affect the physicochemical properties of the experimental resin composites, and ^2^ Exothane-24 would have no effect on the gap formation and ^3^ the polymerization shrinkage stress of the experimental resin composites.

## Materials and Methods

### Resin composite formulation

The control resin composite matrix was formulated with a blend of 50 wt.% Bis-GMA (Sigma Aldrich, St. Louis, MO, USA) and 50 wt.% TEGDMA (Sigma Aldrich, USA). For each experimental group, 10, 20, 30, and 40 wt.% of the BisGMA/TEGDMA blend were respectively replaced with Exothane-24 (Esstech Inc., Essington, PA, USA), as described in Table 1. The photo-initiator system - 0.25 wt.% camphorquinone (Sigma Aldrich, USA) and 0.50 wt.% ethyl-4-dimethylamino benzoate (Sigma Aldrich, USA) - and the photo-polymerization inhibitor - 0.01 wt.% 2,6-bis(1,1-dimethylethyl)-4-methylphenol (Sigma Aldrich, USA) - were then added to each matrix (control and experimental).

The control and the four experimental resin matrixes were loaded with 65 wt.% of silanized filler particles: of these 20 wt.% were 0.05 μm fumed silica (Nippon Aerosil Co. Ltd., Yokkaichi, Tokyo, Japan) and 80 wt.% of 0.7 μm BaBSiO_2_ glass (Esstech Inc., Essington, PA, USA). The experimental materials were mechanically blended (SpeedMixer, DAC 150.1 FVZ-K, Hauschild Engineering, Hamm, North Rhine-Westphalia, Germany).

**Table 1:** Main matrix composition (wt.%) in each experimental resin composite group.

Groups	BisGMA	TEGDMA	Exothane-24
Control	50	50	0
Exothane 10%	45	45	10
Exothane 20%	40	40	20
Exothane 30%	35	35	30
Exothane 40%	30	30	40

### Ultimate tensile strength (UTS)

Hourglass-shaped specimens (10 mm long × 2 mm wide × 1.5 mm thick) of each experimental resin composite (n = 10) were prepared using rubber molds (constriction area: 1.5 × 1.5 mm; cross-sectional area: 2.25 mm²). They were light-cured for 20 s (Bluephase G2, Ivoclar Vivadent, Schaan, Liechtenstein - irradiance ≈ 1200 mW/cm^2^) and dry stored in light-proof containers at 37 ºC for 24 h. Specimens were fitted in a test jig device and submitted to tensile strength testing in a mechanical testing machine (OM100, Odeme Dental Research, Luzerna, SC, Brazil) at 0.75 mm/min. The UTS was calculated in MPa using the formula: CS = F/A, where **F** was the tensile strength (N) and **A** was the sample’s transversal cross-sectional area (mm^2^).

### Flexural strength (FS) and flexural modulus (FM)

A stainless-steel mold was used to fabricate bar-shaped specimens (n = 10; 25 mm long × 2 mm wide × 2 mm thick) of each experimental resin composite, light-cured for 20 s (Bluephase G2, Ivoclar Vivadent - irradiance ≈ 1200 mW/cm^2^), according to ISO 4049:2019 international standard. Due to the length of the specimens, light-curing was performed in seven overlapping irradiation cycles since the tip of the light-curing unit was about 10 mm wide. The same procedure was done on the other side of the specimen. The specimens were then removed from the molds and stored in light-proof containers containing water at 37 ºC for 24 h. The three-point bending test was carried out in a universal testing machine (Instron, Canton, USA - span between supports = 20 mm) at a crosshead speed of 0.5 mm/min. The load was applied on the specimen side that did not face the tip of the light-curing unit.

The maximum fracture load for the specimens was recorded and the FS was calculated using the following equation:

$$FS\;=\;3FL/(2bh^{2})$$

where: **F** is the maximum load (N) exerted on the specimen; **L** is the distance (mm) between the supports; **b** is the width (mm) of the specimen measured immediately before the test; and, **h** is the height (mm) of the specimen measured immediately before the test.

The elastic and plastic portions in the stress/strain plot generated by the Bluehill 2 software (Instron Corporation, Norwood, MA, USA) were considered to calculate FM using the following equation: FM = L_1_S^3^10^-3^/4BH^3^D, where: **L**_1_ = load (N); **S** = distance between the supports (mm); **B** = width (mm); **H** = height (mm); and **D** = displacement (mm).

### Knoop Hardness (KH)

A rubber mold was used to fabricate disc-shaped specimens (n = 10), 2 mm in thickness × 6 mm in diameter. They were light-cured for 20 s (Bluephase G2, Liechtenstein - irradiance ≈ 1200 mW/cm^2^), removed from the mold, and dry-stored in lightproof containers at 37 ºC for 24 h. The surface facing the light-curing unit was then wet-polished with 1200-grit SiC grinding paper (Buehler, Lake Bluff, IL, USA). An indenter (HMV-2, Shimadzu, Tokyo, Japan) was used for the Knoop hardness readings - five times for each specimen - under a load of 490 N, for 15 s. The average of the five readings was established as the KH mean value for each specimen.

### Hardness reduction (HR) after chemical softening

After the KH measurements, the same disc-shaped specimens (n = 10) were stored in 100% ethanol (Sigma-Aldrich Inc, St Louis, MO, USA) for 24 h and then submitted to the chemical softening test to verify the HR ^9^. After the softening test, the same surface was again tested for KH - five readings for each specimen. The HR was defined by the reduction in the KH values after ethanol storage and expressed in percentage. The average of the five readings was established as the HR mean value for each specimen.

### Degree of conversion (DC)

Fourier transform infrared spectroscopy (FT-IR) was used to measure the DC of each experimental resin composite. Five resin composite discs (7 mm in diameter × 2 mm thick) were made in rubber molds and light-cured for 20 s (Bluephase G2, Liechtenstein - irradiance ≈ 1200 mW/cm^2^). Each specimen was held onto the FT-IR chamber (Nicolet Nexus 6700, Thermo Scientific, Waltham, MA, USA). The absorption spectra of the polymerized and non-polymerized resin composites were recorded (acquisition time: 10 sec) in a range between 1500 and 1800 cm^-1^. Three successive measurements in distinct points were considered to determine the average spectrum value. DC was calculated by estimating the changes in the peak height ratio (R) of the absorbance intensities of the aliphatic C=C peak at 1638 cm-1 and that of an internal standard peak of the aromatic C=C at 1608 cm-1 during polymerization, using the following equation:

$$DC\;\left(\%\right)=\;100\;\times [1\;-\;\left(R\;polymerized\frac{}{}R\;non-polymerized\right)]$$

### Gap width (GW)

Inner-polished metallic molds were used to fabricate disc-shaped specimens (n = 8), 2 mm in thickness × 6 mm in diameter, of each resin composite. No adhesive was applied to the mold walls before the insertion of the resin composites. After light-curing (Bluephase G2, Liechtenstein - irradiance ≈ 1200 mW/cm^2^), the specimens were kept in the metallic molds and dry-stored in lightproof containers at 37 ºC for 24 h. The top surface of each specimen was then polished with 320, 400, 600, and 1200-grit SiC grinding paper (Buehler, Lake Bluff, IL, USA).

After polishing, the specimens were mounted in stubs. The width of the gap formed between the metallic mold and the resin composite was measured using scanning electron microscopy (SEM) at low vacuum (back-scattered electrons, LEO 435 VP, Cambridge, England) and a magnification of 1500x, using the SEM software (LEO 435 VP). The measurements, expressed in micrometers, were taken in four positions corresponding to 3, 6, 9, and 12 hours of a clock face. The readings were carried out on the specimen side that faced the light-curing unit. The average arithmetic value of the four readings was established as the mean value of each specimen ^10^.

### Polymerization Shrinkage Stress (PSS) simulating the compliance of Class I (SS-I) and Class II (SS-II) cavities

The PSS was analyzed using a universal testing machine ^6^. Two glass rods (13 and 54 mm in length × 4 mm in diameter) had their contact ends roughened with #180-grit SiC grinding paper and treated with a silane agent (Monobond S, Ivoclar Vivadent, Liechtenstein), while the other end of the 13-mm rod was polished to avoid light transmittance interference. The 54-mm rod was held into an upper fixture, attached to the load cell of a universal testing machine (Instron 4411, Instron, Canton, MA, USA). The 13-mm lower rod was held into a stainless-steel fixture, consisting of a slot that allowed the light-curing tip to access the fixture aperture, through which the curing light reached the resin composite - 1 mm in thickness - lying between the glass rods.

A video extensometer was positioned perpendicular to the composite specimen to measure its shrinkage (µm) through analysis of images taken after and before the light-curing. The video extensometer consists of a digital camera (Nikon D3400, Nikon, Tokyo, Japan) coupled with a macro lens (Nikkor 85 mm, Nikon, Japan) to take images that are analyzed through software (Trackmate, Fiji, ImageJ, National Institute of Health, Bethesda, MD, USA).

The compliance of the shrinkage-measuring apparatus was calculated (1.66 µm/N; C-factor of 0.5) to correct nominal stress values. The light transmittance through the 13 mm rod was 77%; thus, the exposure time was set at 25 s to achieve an energy density of 24 J/cm^2^ (Bluephase G2, Liechtenstein - irradiance ≈ 1200 mW/cm^2^). The resin composite was placed between the treated surfaces of the glass rods and light-cured as the camera captured the images for shrinkage analysis. Ten minutes after light-curing, the strain values obtained from the video recording were manually inputted into the universal machine feedback system and analyzed considering two different compliance situations: 0.4 µm/N (representing Class I cavity) and 3 µm/N (representing Class II cavity). ^11^

$$Nominal\;Polymerization\;Stress\;\left({PS}_{nominal}\right)={Force}_{universal\;testing\;machine\;(N)}$$

$$Corrected\;Polymerization\;Stress\;\left({PS}_{corrected}\right)=\left[Strain\;\left(µm\right)\;x\;\frac{{Compliance}_{apparatus}}{{Compliance}_{cavity}}\right]$$

The formula to calculate the sum of nominal and corrected polymerization stress forces was based on a previous study ^12^. The maximum polymerization stress was calculated by summing the PS_nominal_ and PS_corrected_ and dividing the maximal force by the cross-sectional area of the glass rod. Five specimens (n = 5) were tested for each experimental group.

### Statistical Analysis

The data obtained from the experimental tests (UTS, FS, FM, H, HR, DC, GW, SS-I, and SS-II) were first submitted to Shapiro-Wilk’s and Lavene’s to analyze data normality and homoscedasticity. Then 1-way ANOVA was performed, and the independent variables were set as the resin composite formulations (10% of Exothane-24, 20% of Exothane-24, 30% of Exothane-24, 40% of Exothane-24, and control). Tukey’s test was applied to compare the mean values among the groups (α = 0.05).

## Results

Table 2 shows the mean values and standard deviation concerning the UTS, FS, and FM tests - control and the experimental groups. About UTS and FS, no significant difference (UTS - p = 0.7411; FS - p = 0.1466) was observed among the groups. The control showed the highest FM mean value, statistically different from those obtained for Exothane 30% and Exothane 40% (p = 0.0398).

**Table 2:** Ultimate tensile strength (UTS), flexural strength (FS), and flexural modulus (FM) mean values (standard deviation) for the control and experimental resin composites.

Groups	UTS (MPa)	FS (MPa)	FM (GPa)
Control	47.08 (13.33) a	117.99 (20.93) a	2.23 (0.30) a
Exothane 10%	42.54 (9.49) a	129.58 (27.37) a	1.91 (0.41) ab
Exothane 20%	40.76 (11.36) a	128.36 (15.31) a	1.87 (0.38) ab
Exothane 30%	42.71 (8.23) a	133.90 (11.45) a	1.56 (0.28) b
Exothane 40%	42.81 (10.60) a	120.22 (22.54) a	1.44 (0.31) b

Different letters in each column indicate statistical differences.

Table 3 shows the mean values and standard deviation for H, HR, and DC concerning the control and the experimental resin composites. With regard to H, the control showed the highest mean values and differed from the experimental groups (p = 0.00001), among which Exothane 10% and 20% showed the highest mean values, statistically different from Exothane 30% and 40%. In relation to HR, the control showed the highest mean value and differed from the experimental resin composites (p = 0.00001), among which Exothane 40% and 30% showed the lowest mean values and statistically differed from Exothane 10%. Exothane 20% showed no significant difference from Exothane 30% and differed from the other experimental groups. About DC, the control, Exothane 10%, and 20% showed the highest mean values and differed from Exothane 40% (p = 0.00001). No statistically significant difference was observed among Exothane 10%, 20%, and 30%.

**Table 3:** Hardness (H), hardness reduction (HR), and degree of conversion (DC) mean values (standard deviation) for the control and experimental resin composites.

Groups	H (KHN)	HR (%)	DC (%)
Control	47.10 (2.98) a	19.92 (2.45) a	64.11 (3.71) a
Exothane 10%	34.07 (3.27) bc	15.16 (2.41) b	62.78 (3.88) ab
Exothane 20%	36.75 (2.57) b	10.76 (1.97) c	61.30 (2.79) ab
Exothane 30%	32.61 (3. 21) c	7.95 (2.30) cd	57.25 (3.06) bc
Exothane 40%	28.44 (1.55) d	6.47 (1.66) d	54.74 (2.94) c

Different letters in each column indicate statistical differences.

Table 4 shows mean values and standard deviation for GW, SS-I, and SS-II concerning the control and experimental resin composites. With regard to GW, the control showed the highest mean value and differed from the experimental groups (p = 0.00001), among which no significant difference was observed. In relation to SS-I, the control showed the highest mean value and differed from the experimental groups (p = 0.00002), among which Exothane 30% and 40% showed the lowest mean values and statistically differed from Exothane 10%. Exothane 20% showed no significant difference among the experimental groups. As for SS-II, the control also showed the highest mean value and differed from the experimental groups (p = 0.00074), among which no significant difference was observed.

**Table 4:** Gap width (GW) and polymerization shrinkage stress in simulated compliance of Class I (SS-I) and Class II (SS-II) mean values (standard deviation) for the control and experimental resin composites.

Groups	GW (μm)	SS-I (MPa)	SS-II (MPa)
Control	33.31 (5.60) a	16.13 (1.46) a	3.12 (0.87) a
Exothane 10%	21.36 (4.25) b	6.64 (0.89) b	1.60 (0.45) b
Exothane 20%	18.23 (5.54) b	4.55 (1.22) bc	1.34 (0.15) b
Exothane 30%	18.45 (4.90) b	3.98 (0.47) c	1.08 (0.25) b
Exothane 40%	13.18 (3.59) b	3.64 (0.35) c	0.90 (0.55) b

Different letters in each column indicate statistical differences.

## Discussion

Previous studies have investigated the effect of elastomeric urethane monomers, such as Exothane^™^, on the properties of resin-based materials ^6^ ^,^ ^7^ ^,^ ^13^. Although previous attempts to use elastomeric urethane monomers in formulations of dental adhesive systems resulted in unsatisfactory outcomes ^7^, the chemical characteristics of the monomer Exothane-24 showed promising results for use in monomeric matrices of resin composites ^6^ ^,^ ^13^. However, this is the first study to comprehensively demonstrate the effects of Exothane-24 concentrations on the PSS in different compliance systems and HR after chemical softening. The first null hypothesis that Exothane-24, at any of the concentrations tested, would not affect the physicochemical properties of the experimental resin composites was rejected.

Despite its lower DC, Exothane-24 is the hardest in the Exothane monomer family ^7^, and with an elongation close to that of the monomers UDMA, Bis-EMA, and Bis-GMA ^7^, a property considered fundamental for the formulation of a resin composite with elastomeric characteristics. The very discrepant elongation of the methacrylate monomers could alter the viscoelasticity of the resin composite and thus affect its resilience and elasticity modulus, making it very elastic, which is an undesirable property for dental restorative materials ^14^. The most important clinically relevant finding was that Exothane-24 improved the HR of the experimental resin composites. Previous studies have demonstrated that polymer crosslinking is highly associated with polymer resistance to degradation ^15^ ^,^ ^16^. The Exothane-24-containing resin composites’ resistance to chemical softening might be associated with its molecular structure with four polymerizable groups and the elastomeric ether ligand structures that increase polymer cross-linking. Also, the urethane (-NH) molecular structure can improve the chain transfer and also increase the polymer cross-linking ^17^. A previous study ^6^ reverse-engineered the molecular structure of the Exothane-24 and demonstrated that composites with Exothane-24 have higher wear resistance than regular urethane dimethacrylate monomers. Still, further investigations need to evaluate the dynamic mechanical properties of composites containing Exothane-24.

However, the higher polymer cross-linking from a tetra-functional methacrylate monomer comes with a drawback. More functional groups available would lead to more double bonds available for the polymerization, but the DC is calculated by the ratio of the aliphatic double bonds available before and after curing. Despite the lower DC of Exothane-containing resin composites, the number of double bonds formed during the polymerization of tetra-functional methacrylate monomers is theoretically higher than mono or di-methacrylate monomers ^6^. A previous study ^13^, failed to address that a lower DC does not mean fewer double bonds were formed and this affirmative can be explained by the fact that the study has not found differences in the mechanical properties of the composites containing Exothane-24. Exothane-24, at all concentrations tested, influenced the H and HR values (Table 3) compared with those of the control. The lower H values observed might be related to the flexural modulus of resin composites containing Exothane-24 ^19^. It can be inferred that the lower the flexural modulus of the material, the lower its stiffness, facilitating penetration of the indenter during the hardness test, although this reasoning was valid only for concentrations of 30 and 40% of Exothane-24.

The lower HR values obtained for the composites containing Exothane-24, when compared with those recorded for control, suggest that, despite the lower DC, a higher density of cross-links when the Exothane-24 is present, indicating better quality of the polymer chains formed (9, 20). Thus, the first null hypothesis is rejected, where it is stated that the inclusion of Exothane-24 would not affect the physicochemical properties of the composite resin.

Exothane-24, at any of the concentrations tested, did not affect the UTS and FS values compared with those of the control (no Exothane-24), except for FM values, which decreased significantly with Exothane-24 at 30% and 40% (Table 2). This decrease in FM might be associated with the low stiffness of the formed chains ^18^. The FS test consists of tensile and compression stresses (resulting in shear) while, in the UTS test, tensile stress occurs predominantly. In the FS tests, Exothane-24, at any concentration, had no influence on the stress values due to the complexity of the stresses involved.

When compared to the control, Exothane-24, at all concentrations, reduced GW significantly; no statistical difference was observed among the concentrations (Table 4). This finding may be associated with the high molecular weight of Exothane-24 (682.85 g/mol), and the slightly lower DC obtained for the composites containing Exothane-24, conditions that might reduce the volumetric contraction and, consequently, the gap width. The viscosity of Exothane-24 (6 Pa·s) is known to lie between those of Bis-GMA (1200 Pa·s) and TEGDMA (0.01 Pa·s) and can also contribute to these results ^8^. Thus, the second null hypothesis - Exothane-24 would not affect the GW - was rejected.

The third null hypothesis - Exothane-24 would not affect the PSS (SS-I and SS-II) of the composites during polymerization - was also rejected. This reduction in PSS might be related to the Exothane-24’s molecular structure, which differs from BisGMA and TEGDMA. According to the CHN elemental analysis, Exothane-24 has a molecular weight (682.85 g/mol) higher than those of BisGMA (512 g/mol) and TEGDMA (286 g/mol). ^19^ These molecular weight differences might have affected the volumetric polymerization shrinkage, decreasing the polymerization SS of the experimental resin composites. Besides, Exothane-24 has polyol and cycloaliphatic core structures that are more prone to stretch and adapt in stress-developing scenarios ^21^. It also has four polymerizable functional groups, which may lead to stress-strain dissipation, binding, and crosslinking during polymerization ^22^ ^,^ ^23^.

Importantly, the PSS was measured in two compliance situations, Class I (SS-I) and Class II (SS-II) simulated cavities. As observed, Exothane-24 significantly influences the stress developing in systems with low compliance, such as Class I cavities. An implication of this is the possibility that resin composites containing Exothane-24 might develop less stress in the bonding interface and consequently increase the bond strength to dentin and reduce the gap formation. For Class II cavities with higher compliance, the resin composites containing Exothane might reduce the stress on the buccal and lingual cusp in the proximal box, reducing the cusp deflection and fatigue, which reduces the risk of tooth fracture.

The composites containing Exothane-24 at 30 and 40% concentrations showed lower FM mean values (Table 2) compared to those of the control in both system compliance situations (SS-I and SS-II). Overall, FM is equal to the ratio of stress and strain; the lower the FM, the lower the stress for a given strain ^24^. Given that, composites with lower FM might have lower polymerization stress ^25^. Accordingly, the lower polymerization shrinkage stress mean values (Table 4) recorded for the resin composites containing Exothane-24 might be associated with their lower FM.

The lower DC mean values (Table 3) of the resin composites containing Exothane-24 might be associated with the reduction in PSS ^25^. Further studies applying bond strength tests using box-shaped tooth cavities (high C-factor) are needed to verify our findings. A different bond strength test to assess the effect of the polymerization PSS on adhesive interfaces could complement our results and help us understand the shrinkage stress in constrained situations.

In conclusion, Exothane-24 at concentrations of 20% and 30% reduced the gap width, and polymerization shrinkage stress of the experimental resin composites tested without affecting their physicochemical properties, except for the Knoop microhardness and flexural modulus (only 30%). Therefore, Exothane-24, at these concentrations, seems to be a promising monomer for the formulation of resin composites aiming at reducing their polymerization shrinkage stress without significantly impairing any other physicochemical properties.
